# A national nudge study of differently framed messages to increase COVID-19 vaccine uptake in Saudi Arabia: A randomized controlled trial

**DOI:** 10.1016/j.jsps.2023.101748

**Published:** 2023-08-15

**Authors:** Mohammed Alhajji, Abdullah H. Alzeer, Rami Al-Jafar, Reem Alshehri, Saad Alyahya, Sara Alsuhaibani, Sarah Alkhudair, Raghad Aldhahiri, Ahmed Alhomaid, Dalal Alali, Abdulelah Alothman, Eman Alkhulaifi, Mohammed Alnashar, Abdulrahman Alalmaee, Ibrahem Aljenaidel, Fahad Alsaawi

**Affiliations:** a Nudge Unit, Ministry of Health, Riyadh, Saudi Arabia; b Data Services Sector, Lean Business Services, Riyadh, Saudi Arabia; c Department of Clinical Pharmacy, College of Pharmacy, King Saud University, Riyadh, Saudi Arabia; d School of Public Health, Imperial College London, London, UK; e Department of Health Sciences, College of Health and Rehabilitation Sciences, Princess Nourah bint Abdulrahman University, Riyadh, Saudi Arabia; f E-Services, Lean Business Services, Riyadh, Saudi Arabia; g Process Optimization, Lean Business Services, Riyadh, Saudi Arabia

**Keywords:** COVID 19 Vaccine, Reminders, Nudge, Framing, In-App notification

## Abstract

**Background:**

During the COVID-19 pandemic, Saudi Arabia witnessed hesitancy from a proportion of the population toward taking the vaccine; thus, it was necessary to nudge them to uptake it. This study was conducted to assess the impact of using different types of messages to nudge the public to increase the proportion of vaccinated individuals.

**Methods:**

This study is a multi-arm randomized controlled trial aiming to assess the efficacy of using differently framed messages that appear as pop-notifications in Sehatty application. Of those who preregistered to receive a COVID-19 vaccine but didn’t take it according to the Saudi national vaccine registry (n = 1,291,686), 12,000 individuals were randomly recruited and randomly assigned to one of five intervention groups (commitment, loss aversion, salience, social norms, and ego) or a control group. To ensure the exposure occurred in the intervention groups, we included only those who received the notification, which was confirmed by checking the information technology system. We used the Chi-square test to compare each intervention group against the control group separately. Also, we used the same test to investigate whether sex and age influenced the percentage of booked appointments in the intervention groups.

**Results:**

Social norms, ego, salience and loss aversion groups had higher percentages of booked appointments when compared to the control group (21.0%, *p* = 0.001; 19.1%, *p* = 0.011; 19.0%, *p* = 0.013; 18.4%, *p* = 0.034, respectively). Moreover, when combining the intervention groups, the percentage was higher than the control group (*p* < 0.001). The percentages of booked appointments made by Young adults (18–35 years old) were higher than that of adults over 35 years old in the social norms (22.6%, *p* = 0.016) and ego groups (21.0%, *p* = 0.010). At the same time, sex didn’t affect the percentages of booked appointments in any group.

**Conclusion:**

Using different framings of messages to nudge the public to take vaccines can help increase the percentage of immunized individuals in a community. Nudges can boost the public health of a population during an unusual spread of vaccine-preventable diseases. Findings might also inspire governmental responses to other public health situations.

## 1 Introduction

In the last quarter of 2019, the severe acute respiratory syndrome coronavirus 2 (COVID-19) emerged and spread around the globe (Li et al., 2020). To help control the impact of this massive global health challenge (Dhama et al., 2020, He et al., 2021), the World Health Organization (WHO) declared COVID-19 a pandemic in March 2020 (World Health Organization, 2020), and governments moved swiftly to implement policies (Phan and Narayan, 2020) suggested by WHO and local health agencies (Alsayedahmed, 2020). These urgent precautions included lockdown of public services (Di Domenico et al., 2020), imposing a curfew, mandating quarantine, social distancing (Ciotti et al., 2020), and travel restrictions (Chinazzi et al., 2020). While such measures assisted in limiting the number of new cases and slowed the spread of the virus (Almutairi et al., 2020), they engender tremendous unanticipated socio-economic and political consequences (Yezli and Khan, 2020).

In Saudi Arabia, the authority took advanced measures by providing different variants of the vaccine for all citizens and residents through a platform called “Sehhaty” (a smartphone application that aims to enable Saudi citizens to reach health information and medical e-services provided by different health organizations in the Kingdom) (Saudi Ministry of Health, 2023). Vaccine appointments could be reserved easily through the application and accessed almost everywhere.

During the first several months of COVID-19 vaccination availability in Saudi Arabia, they were perceived as unsafe and unnecessary by a large number of individuals (Dubé et al., 2013), which could impose personal risks and hinder herd immunity (Jacobson et al., 2015). Such hesitancy threatens global health and could lead to outbreak reemergence (Dai et al., 2021). Thus, evidence-based strategies that could be rapidly deployed at scale to encourage vaccination were urgently needed. Such strategies can be informed by behavioral sciences, which are typically low-cost and scalable (Milkman et al., 2021)^.^

Behavioral science is currently utilized in understanding the reasons for vaccination acceptance and hesitancy (Lorini et al., 2020). It could be utilized to increase vaccination rates using an array of behavioral interventions, such as “nudges” (Glanz and Bishop, 2010, Olstad et al., 2014). Nudge interventions function by changing the “choice architecture” through which the individual makes decisions. In addition, nudges operate mainly by influencing individual’s inherent cognitive biases, a process that can encourage a socially desirable behavior without any limitations in choice (Gold et al., 2023, Patel et al., 2018, Sant’Anna et al., 2021). Nudges can be designed to remind, guide, or motivate behavior (Patel et al., 2018) by applying various techniques such as defaults, alerts, peer comparison, information transparency, framing, and accountable justification (Sant’Anna et al., 2021).

Some of the nudge’s strategies have been shown to be influential with significant effects and results (Hummel and Maedche, 2019). For instance, a nudge unit in the United Kingdom increased organ-donor consent rates (Patel et al., 2018), increased tax revenues and charitable contributions (Patel et al., 2018), and increased generic prescribing rates from 75% to 98% using only nudge methods (Patel et al., 2016). Such an approach is used in improving civic behaviors (John et al., 2009) and governmental regulations worldwide (Patel et al., 2018). Although these nudges could be beneficial to apply during the pandemic to increase the number of vaccinated individuals in the Saudi population, they have not been utilized yet in Saudi Arabia. This study aims to examine the efficacy of five types of framing on nudging Saudi citizens to take the COVID-19 vaccine. The study could inspire governmental responses to contain vaccine-preventable diseases.

## 2 Methodology

### 2.1 Design and setting

This multi-arm randomized controlled trial examined the efficacy of the following five types of messages in nudging citizens to take the COVID-19 vaccine: commitment, loss aversion, salience, social norms, and ego. The intervention was a pop-up message on Sehhaty application (Ministry of Health personal medical record app).

### 2.2 Population, sampling, intervention and outcome

The study population includes individuals 18 and older who have previously installed Sehhaty and preregistered for COVID-19 vaccination, but never had any documented COVID-19 vaccination on the national vaccine registry (NVR) in Saudi Arabia. Illegible individuals for vaccination were excluded. Individuals who had a positive COVID-19′s PCR test within the last ten days before starting the study were also excluded. Moreover, nationalities other than Saudi were excluded to properly capture the local and cultural nuances in the Arabic language. Our inclusion/exclusion criteria yielded 1,291,686 eligible individuals. Then, 12,000 (∼1%) were randomly selected from the targeted population stratified for sex and age groups.

**Fig. 1:**
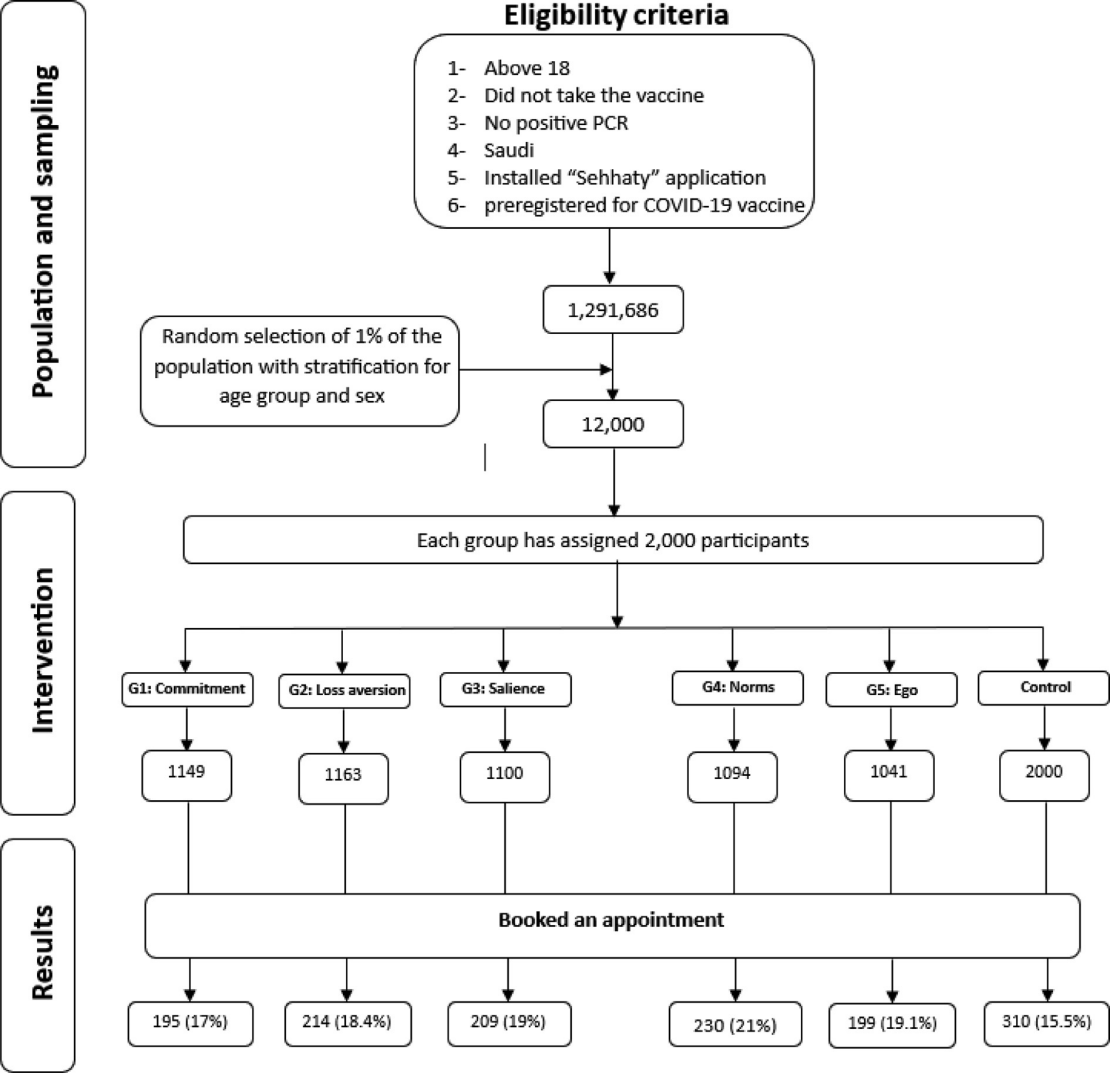
The study protocol and results summary.

Eligible individuals were randomly and equally assigned to one of five intervention groups or a control group matched for age group and sex. Each intervention group received one type (commitment, loss aversion, salience, social norms, or ego). And to ensure the elimination of technical barriers that might affect the study results, the intervention groups included only those who indeed received the notification were included in the analyses (checked in the backend of the information technology system operating the app). Fig. 1 summarizes the study protocol.

These messages were framed based on the MINDSPACE framework, which outlines the most common forces that affect human behavior: Messenger, Incentives, Norms, Defaults, Salience, Priming, Affect, Commitment, and Ego (Dolan et al., 2012). This behavioral change framework based on behavioral economics and psychology has been used numerous times in behavioral science interventions to influence decision-making in a different context at a marginal cost (Dolan et al., 2012, Reñosa et al., 2021). Messages had undergone thorough delineation and multiple revisions from the research team at the nudge unit to ensure cultural sensitivity and linguistic accuracy. To remove any frictions that could hinder participants from booking their appointment to get the vaccine, the pop-up messages concluded with a direct link to book the vaccine appointment (the link is universal for all types of smartphones to ensure inclusivity). Adding the booking link ensured directing participants to the desired behavior immediately after reading the message, the cue. These messages appeared as a pop-notification on participant’s smartphone. Only those who indeed received the notification were included in the analyses (checked in the backend of the information technology system operating the app). The control group did not receive any messages or alerts. The content of each message is listed in Table 1.

**Table 1:** Intervention message scripts.

**Massage type**	**Original script**	**Translated script**
**Commitment**	استكمالًا لخطوات حصولك على لقاح كورونا، احجز جرعتك الأولى خلال 48 ساعة القادمة عبر تطبيق صحتي	To finish your steps in getting the COVID vaccine, book your first dose in the next 48 h through Sehhaty: [direct link]
	https://identity.sehhaty.com/download	
**Loss aversion**	لا تكن ضحية فيروس كورونا المتحوّر ذو الانتشار السريع والأعراض الخطيرة. احجز جرعتك الأولى الآن عبر تطبيق صحتي	Do not be a victim of COVID newest variant with its fast spread and threatening symptoms. Book your first dose now through Sehhaty: [direct link]
	https://identity.sehhaty.com/download	
**Salience**	أغلب مرضى كورونا الموجودين في العناية المركزة هم أشخاص لم يأخذوا اللقاح. احجز جرعتك الأولى الآن عبر تطبيق صحتي:s	Most COVID patients in the ICU are those who did not take the vaccine. Book your first dose now through Sehhaty: [direct link]
	https://identity.sehhaty.com/download	
**Norms**	حسب بيانات وزارة الصحة، فإنك من الفئة القليلة التي سجلت للقاح ولم تأخذه حتى الآن. مناعتنا المجتمعية بيدك. احجز جرعتك الأولى عبر تطبيق صحتي	Data from the Ministry of Health indicates you are among the few who preregistered to get vaccinated but haven’t yet. Our immunity is on your hands. Book your first dose now through Sehhaty: [direct link]
	https://identity.sehhaty.com/download	
**Ego**	افتخر بدورك في الحفاظ على صدارة المملكة عالميًا في مواجهة الجائحة. أخذك للقاح يحميك ويبقينا في القمة. احجز جرعتك الأولى عبر تطبيق صحتي	Take pride of your role in keeping the kingdom as a global leader in fighting the pandemic. Taking the vaccine protects you and keeps us on the top. Book your first dose now through Sehhaty: [direct link]
	https://identity.sehhaty.com/download	

The main outcome was whether the participant had made a vaccination appointment by the end of the fifth day after receiving the message. Appointment status was captured through the national vaccine registry. All messages were sent in the afternoon on the first day of the week (Sunday in Saudi Arabia). The intervention was concluded by the end of the fifth day of the week (Thursday) between Sep 5th-9th 2021.

The Institutional Review Board in the Saudi Ministry of Health approved the study (IRB Log No: 21–105 E) and waived the requirement for consent.

### 2.3 Analysis

Chi-square test was used to evaluate the efficacy of the intervention, comparing each group separately with the control group. The outcome variable, making a vaccine appointment, was dichotomous (booked or not booked). Ancillary analyses were conducted to examine sex and age differences in the intervention groups using Chi-square. All analyses were conducted using SPSS v. 23. Statistical significance was set at *p* < 0.05.

## 3 Results

The study included 7,547 participants, mostly females (60.4%). Younger individuals comprised more than half of the study sample (62.1%) (Table 2).

**Table 2:** Description of sample’s demographics.

**Variable**		**N = 7,547**	**%**
**Sex**	Female	4,561	60.4
	Male	2,986	39.6
**Age groups**	18–25	2,066	27.4
	26–35	2,619	34.7
	36–45	1,618	21.4
	46–55	671	8.9
	56–65	463	6.1
	>65	110	1.5

Comparing the rate of booked appointments in each intervention group with that of the control group showed all framings except commitment significantly increased the rate of booked appointments (Table 3). Specifically, the social norms framing group had the highest significant increase in the percentage of booked appointments (21.0%, *p* = 0.001), followed by the ego group (19.1%, *p* = 0.011), and the salience group (19.0%, *p* = 0.013) as compared individually against the control group. When comparing the control group with the combined intervention groups, the difference was significant, where 18.9% of individuals in the intervention arm booked appointments compared to 15.5% in the control arm (*X*^2^ = 11.40; *p* < 0.001) (Table 3).

**Table 3:** The Chi-square test comparing the percentage of booked appointments between control group and intervention groups.

**Variable**	**Appointment Booked**		***X*^2^**	***p***
	**Yes**	**No**		
**Commitment**	195 (17.0%)	954 (83.0%)	1.17	0.279
**Control**	310 (15.5%)	1,690 (84.5%)		
**Total**	505 (16.0%)	2,644 (84.0%)		
**Loss Aversion**	214 (18.4%)	949 (81.6%)	0.04	0.034
**Control**	310 (15.5%)	1,690 (84.5%)		
**Total**	524 (16.6%)	2,639 (83.4%)		
**Salience**	209 (19.0%)	891 (81.0%)	−0.05	0.013
**Control**	310 (15.5%)	1,690 (84.5%)		
**Total**	519 (16.7%)	2,581 (83.3%)		
**Norms**	230 (21.0%)	864 (79.0%)	−0.07	0.001
**Control**	310 (15.5%)	1,690 (84.5%)		
**Total**	540 (17.5%)	2,554 (82.5%)		
**Ego**	199 (19.1%)	842 (80.9%)	−0.05	0.011
**Control**	310 (15.5%)	1,690 (84.5%)		
**Total**	509 (16.7%)	2,532 (83.3%)		
**Intervention**^a^	1,047 (18.9%)	4,500 (81.1%)	11.40	<0.001
**Control**	310 (15.5%)	1,690 (84.5%)		
**Total**	1,357 (18.0%)	6,190 (82.0%)		

a : Intervention groups combined.

Ancillary analyses were conducted to assess sex and age differences in appointments booked by intervention groups. To dichotomize age, young adult refers to those 18–35 years old (n = 4,685), and older adults refer to those 55 and older (*n* = 1,244). There was no difference in the rate of booked appointments made by young adults compared to those made by older adults in commitment, loss aversion, and salience groups. However, the rate was higher amongst young adults in the social norms and ego groups (22.6%, *p* = 0.016; 21.0%, *p* = 0.010, respectively). When we combined the intervention groups, the percentage of booked appointments by young adults was more pronounced compared to that made by older adults (20.1% vs 15.5%, *X*^2^ = 9.10; *p* = 0.003) (Table 4).

**Table 4:** The Chi-square test comparing the percentage of booked appointments by age groups.

**Variable**		**Group**	**Appointment Booked**		***X*^2^**	***p***
			**Yes**	**No**		
**Type of Message**	**Commitment**	Younger	126 (17.6%)	591 (82.4%)	0.07	0.797
		Older	30 (16.8%)	149 (83.2%)		
		Total	156 (17.4%)	740 (82.6%)		
	**Loss Aversion**	Younger	141 (19.2%)	592 (80.8%)	0.04	0.850
		Older	32 (18.6%)	140 (81.4%)		
		Total	173 (19.1%)	732 (80.9%)		
	**Salience**	Younger	141 (20.2%)	556 (79.8%)	1.71	0.191
		Older	26 (15.8%)	139 (84.2%)		
		Total	167 (19.4%)	695 (80.6%)		
	**Norms**	Younger	159 (22.6%)	546 (77.4%)	5.75	0.016
		Older	21 (13.8%)	131 (86.2%)		
		Total	180 (21.0%)	677 (79.0%)		
	**Ego**	Younger	141 (21.0%)	532 (79.0%)	6.56	0.010
		Older	19 (12.0%)	139 (88.0%)		
		Total	160 (19.3%)	671 (80.7%)		
	**Intervention**^a^	Younger	708 (20.1%)	2,817 (79.9%)	9.10	0.003
		Older	128 (15.5%)	698 (84.5%)		
		Total	836 (19.2%)	3,515 (80.8%)		

a : Intervention groups combined.

The same analysis in Table 4 was conducted to assess sex differences in outcome (females = 4,561; males = 2,986), but there were no differences related to sex.

## 4 Discussion

This study aimed to assess the efficacy of different nudges in influencing citizens to book an appointment for their first dose of COVID-19 vaccine in Saudi Arabia. The target population were individuals who had already shown interest and preregistered to take the vaccine, yet they had not proceeded with their plans to book an appointment. Given this strong sign of hesitancy, nudge theory was ideal as it is best utilized to address hesitancy and not extreme rejection or complete lack of interest (Giubilini et al., 2019). Our findings in this study are consistent with previous research showing that text-based nudges can influence decision-making in hesitant individuals (Freeman et al., 2021, Lorini et al., 2020, Motta et al., 2021, Reñosa et al., 2021). Results from the current study show all types of messages, except for commitment framing, were effective and showed a significant increase in booking vaccination appointments across different groups. Similar studies have shown that using different frames to deliver messages to the target audience is promising and can increase vaccine intention and uptake (Dai et al., 2021, Lorini et al., 2020, Milkman et al., 2021, Reñosa et al., 2021). Moreover, the percentage of young adults who booked appointments for vaccination after receiving the nudge messages was higher than those over 35 years old.

In this study, the most influential framing was social norms which emphasized that the majority have taken the vaccine. People are typically and predictably influenced by what others are doing due to our deep innate nature to have affiliation with members of similar groups (Agranov et al., 2021, Vlaev et al., 2016). Thus, employing social norm is a powerful force to change behaviors. When norm messages were designed for this intervention, being in the minority was strongly highlighted, accompanied by an emphasis on participants’ role in protecting the community to evoke a sense of responsibility towards the community. Likewise, a study conducted by Agranove et al examined the effect of free riding, social norms, and herding on the decision to take COVID −19 vaccine and found that individuals were positively influenced by others’ decision to vaccinate (Agranov et al., 2021).

Ego and salience framings also demonstrated promising results. People generally lean toward behaving in what supports a positive self-image and perform better when high expectations are placed upon them (Dolan et al., 2012, Santos et al., 2021, Vlaev et al., 2016). Indeed, the ego message in this study emphasized the importance of these individuals in keeping the country in the lead and protecting the community by taking the vaccine. Furthermore, in the age of social media, incessant information overload, and cognitive saturation, attention has become a scarce commodity and a challenge to public health authorities and private sectors alike in what is known as the “attention economy” (United Nations). Therefore, one of the principal nudges is salience which operates by emphasizing certain elements of the structural or informational environment to make said elements more appealing and present in the decision-making process (Noggle, 2018). The current study utilized salience by underscoring the fact that most patients in the ICU are those who had not gotten the vaccine; a fact that was hypothesized to “standout” in the stream of information people receive in their phones.

On the other hand, the commitment framing used in this study did not show a significant effect. Text-based commitment is not utilized extensively in behavior science research due to the complex processes involved in commitment and the need to employ extensive efforts to achieve results, as people are generally reluctant to commit (Dolan et al., 2012). The message involved in this study asked participants to complete the steps of getting COVID-19 vaccine by booking their appointment within 48 h. So, it is possible that providing the urgent time window of 48 h backfired as participants might have other competing priorities and therefore decided to delay acting altogether since they could not fulfil the requirement during the given timeframe.

Additional analysis by age revealed that younger adults (18–35) were significantly more stimulated by the social norms and ego messages than older participants. This contrasts with a study by Milkman and colleagues which did not show any significance among different age groups in their response to different framings (Milkman et al., 2021). The current results are aligned with the evidence that younger generations are more prone to social norms and pressure (Knoll et al., 2017). In addition, the results align well with the current Saudi sociopolitical context, where the sense of nationalism is more heightened than ever in the new era spearheaded by the younger leadership. In fact, one of the most celebrated facts in Saudi today, especially in the political discourse, is that 70% of citizens are 35 and younger, who are the executors and the enablers of the country’s Vision 2030 (General Authority for Statistics, 2020). Therefore, striking the chord of national pride among younger Saudis is a winning card that should be utilized to enhance public health responses.

This study presents several contributions to the literature. Although other studies have shown the effectiveness of salience, social norms, and loss aversion in relation to vaccines uptake (Brewer et al., 2017, Reñosa et al., 2021, Santos et al., 2021), to our knowledge, this is the first study that examined ego or commitment framings in this context. Secondly, the random national sampling, along with randomization and matching of intervention groups, ensures the methodological rigour needed in behavioral sciences and provides stronger external and internal validity. Another strength of the current study is the use of objective measures in determining vaccination status before and after the intervention.

That said, findings should be considered with some limitations. Due to the fragmented information technology infrastructure around COVID vaccine in Saudi, the study protocol could not verify whether participants did in fact take the vaccine, which is the ultimate desired behavior in this context. Here, only the appointment status was assessed. Further investigation can follow up to determine show-up rates, which would further authenticate the power of using MINDSPACE framework for vaccine uptake. In addition, participants’ demographic data were limited to age and sex. It would be helpful to know more about participants’ profiles to enable the formation of media persona and improve message tailoring. Moreover, another limitation is that the study didn’t capture all potential confounding that might contributed to the results of the study.

Nudging techniques are promising when it comes to vaccine uptake, especially when incorporating different approaches (Brewer, 2021, Reñosa et al., 2021). Behavioral science-based interventions have shown effectiveness at a limited cost. For instance, the individual message in this current study cost less than $0.33. Considering the increase reported in the different framings compared to the control group, we urge policymakers to support behavioral sciences-based interventions particularly using norms, ego, and salience in messaging to decrease vaccine hesitancy. Such interventions could be further optimized by making them tailored and targeted to specific demographic groups.

Using nudge interventions is beneficial to decrease vaccine hesitancy, especially among young adults. Nudging hesitant individuals can provide an efficient way to accelerate the pace to control the spread of vaccine-preventable diseases and reduce the need for extreme measures such as lockdowns and their unfavourable associated socio-economic and political consequences.

## Declaration of Competing Interest

The authors declare that they have no known competing financial interests or personal relationships that could have appeared to influence the work reported in this paper.
